# Dynamics of the immune microenvironment and immune cell PANoptosis in colorectal cancer: recent advances and insights

**DOI:** 10.3389/fimmu.2024.1502257

**Published:** 2024-11-29

**Authors:** Jinlong Wan, Jianzhong Zhao, Xiaolu Fang

**Affiliations:** ^1^ Department of Gastroenterology, Gaozhou People’s Hospital, Maoming, China; ^2^ Department of Clinical Laboratory, Xiangyang No.1 People’s Hospital, Hubei University of Medicine, Xiangyang, China

**Keywords:** colorectal cancer, tumor microenvironment, PANoptosis, tumor progression, immune microenvironment

## Abstract

Colorectal cancer (CRC) is one of the most significant oncological threats to human health globally. Patients often exhibit a high propensity for tumor recurrence and metastasis post-surgery, resulting in suboptimal prognoses. One of the underlying reasons for the metastatic potential of CRC is the sustained abnormal state of the tumor immune microenvironment, particularly characterized by the atypical death of critical immune cells. In recent years, a novel concept of cell death known as PANoptosis has emerged. This form of cell death is regulated by the PANoptosome complex and encompasses key features of apoptosis, pyroptosis, and necroptosis, yet cannot be entirely substituted by any of these processes alone. Due to its widespread occurrence and complex mechanisms, PANoptosis has been increasingly reported in various malignancies, enhancing our understanding of its pathological mechanisms, particularly in the context of CRC. However, the characteristics of immune cell PANoptosis within the CRC immune microenvironment have not been thoroughly elucidated. In this review, we focus on the impact of CRC progression on various immune cell types and summarize the distinctive features of immune cell PANoptosis. Furthermore, we highlight the future research trends and challenges associated with the mechanisms of immune cell PANoptosis in CRC.

## 1 Introduction

Colorectal cancer (CRC) is among the most prevalent malignancies globally. According to the latest estimates from the International Agency for Research on Cancer (IARC), CRC ranks third in incidence among all malignant tumors, following lung cancer and female breast cancer (1). Moreover, CRC is the second leading cause of cancer-related mortality globally, underscoring its significant lethality (2–4). Currently, the primary treatment strategy for CRC involves surgical resection combined with adjuvant chemotherapy (5, 6). However, in a subset of patients, tumor recurrence and metastasis frequently occur postoperatively, resulting in suboptimal prognostic outcomes. As a result, identifying and understanding the high-risk factors that influence the prognosis of CRC patients is of paramount importance. A growing body of research indicates that CRC often induces a state of persistent immune dysregulation in the host. Aberrant apoptosis of key immune cells is a critical factor contributing to tumor recurrence and metastasis (7, 8).

Cell death is a fundamental physiological process that occurs in all living organisms, playing critical roles in embryonic development, organ function maintenance, and aging (9, 10). Cell death is typically categorized into two main types: accidental cell death (ACD) and regulated cell death (RCD). Historically, RCD has been regarded as a pivotal mechanism in tumorigenesis (11). However, recent research has unveiled complex interactions among apoptosis, pyroptosis, and necroptosis (12). In 2019, Malireddi et al. introduced a novel concept of cell death termed “pan-apoptosis” (13). Pan-apoptosis is regulated by a pan-apoptotic body complex, encompassing key features of apoptosis, pyroptosis, and necroptosis, but cannot be replaced by any of these processes individually. Given the ubiquity and intricate mechanisms of pan-apoptosis, it has been increasingly reported across various malignancies. A foundational study has indicated that modulation of anti-apoptotic pathways can enhance the sensitivity of cisplatin-resistant laryngeal cancer cells (14). Furthermore, in CRC, phosphorylated cysteine desulfurase has been observed to modulate chemotherapy sensitivity by inhibiting pan-apoptosis (15). The pan-apoptosis of immune cells has also emerged as a significant focus in cancer research.

Therefore, a comprehensive understanding of the pathophysiological mechanisms of pan-apoptosis is crucial for advancing our understanding of CRC occurrence and treatment. Increasing awareness of pan-apoptosis in immune cells associated with CRC provides multiple opportunities to improve therapeutic strategies, including overcoming chemotherapy resistance, reducing treatment side effects, enhancing immune system responsiveness, and ultimately improving treatment efficacy. In this review, we primarily focus on the impact of various immune cell types during CRC development, summarizing the characteristics of pan-apoptosis across these immune cells. Additionally, we further outline future research trends and challenges in elucidating the mechanisms of immune cell pan-apoptosis in CRC.

## 2 Characteristics of immune cell alterations within the immune microenvironment of CRC

The tumor microenvironment (TME) refers to the localized environment surrounding tumor cells (16). This specialized region encompasses not only the tumor cells themselves but also a variety of non-tumor cells, extracellular matrix, blood vessels, lymphatic vessels, and an array of molecular signals (17, 18). The TME plays a pivotal role in tumor progression and metastasis by regulating signals for tumor cell proliferation and survival, reshaping the chronic inflammatory milieu, altering angiogenesis and lymphangiogenesis, and facilitating immune evasion (19, 20). Among these components, immune cells are considered central players in mediating these functions, a perspective that is equally applicable in CRC-TMEs (21). Therefore, a comprehensive understanding of the state of immune cells within the CRC-TME is crucial for effectively modulating the TME, thereby inhibiting CRC metastasis and progression and ultimately improving patient survival rates.

### 2.1 T cell

Tumor-infiltrating lymphocytes (TILs) represent a major component of the immune microenvironment in CRC, primarily comprising T cells and B cells (22, 23). Among these, T cells—including CD8^+^ T cells and CD4^+^ T cells—constitute the most abundant and distinctive immune cells within the tumor immune microenvironment. CD8^+^ T cells, also known as cytotoxic T lymphocytes (CTLs), are a crucial part of the adaptive immune system and serve as the primary effector cells in antitumor immune responses (24). Under normal conditions, CTLs can directly kill tumor cells by recognizing tumor antigens and can further enhance tumor cell lysis by secreting various cytokines (25). However, an increasing number of studies have revealed that CRC employs multiple strategies to disrupt this critical immune response. First, CRC can remodel the TME by mechanisms such as hyaluronic acid accumulation, which hinders the recruitment of CD8^+^ T cells and exacerbates tumor malignancy (26). Additionally, CRC can inhibit CTL cytotoxic activity by expressing immune checkpoint molecules and secreting immunosuppressive cytokines, including interleukin-10 (IL-10) and transforming growth factor-β (TGF-β) (27). Finally, at the epigenetic level, the loss of RNA N6-methyladenosine methyltransferase Mettl14 has been shown to cause dysfunction in CD8^+^ T cells (28).

CD4^+^ T cells can further differentiate into a variety of functionally distinct subpopulations, including helper T cells such as Th1 and Th17 cells, as well as regulatory T cells (Tregs). This process encompasses the intricate regulatory effects of the microenvironment on immune cells, wherein various cytokines play pivotal roles. For instance, interferons facilitate the differentiation of Th1 cells, while interleukin-4 promotes the differentiation of Th2 cells. Moreover, the accumulation of lactate within the tumor microenvironment can inhibit the proliferation and functionality of CD4+ T cells, concurrently fostering the differentiation of Tregs. Furthermore, intercellular interactions significantly influence the transition of CD4+ T cells into distinct subpopulations. Notably, M2 macrophages secrete IL-10, thereby enhancing the formation of Tregs. Overall, this represents a complex and finely-tuned process. These subpopulations play complex roles in the progression and metastasis of CRC, often acting as a “double-edged sword.” For instance, Th17 cells promote tumor growth and metastasis by secreting cytokines like interleukin-17, which suppress the immune characteristics of the microenvironment. Consequently, Th17 cell infiltration is generally associated with poor prognosis. One study analyzing the TME in 125 frozen colorectal tumor specimens found that patients with high expression of the Th17 cluster experienced worse outcomes, whereas those with high expression of the Th1 cluster exhibited prolonged disease-free survival (29). Tregs are thought to play a crucial role in tumor metastasis by inducing “phenotypic plasticity” during tumor spread through the co-expression of pro-inflammatory transcription factors. Beyond their immunoregulatory functions, Tregs have also been implicated in the metabolic adaptation within the CRC-TME In this context, Tregs assist tumor cells in surviving under conditions of nutrient deprivation and hypoxia by regulating metabolic stress, thereby enhancing capacities such as lactate uptake and fatty acid metabolism (30). A study that assessed Tregs by immunohistochemically evaluating the characteristic molecule forkhead box P3 (FOXP3) found that high FOXP3+T regs infiltration was associated with shorter relapse-free survival and disease-specific survival, indicating poorer prognosis for patients with elevated Treg levels (31).

### 2.2 Data collection for proteomics and essential hypertension

In CRC, B cells primarily influence the TME through the secretion of key factors and interactions with tumor cells. Studies have shown that B cells can activate T cells by presenting tumor antigens in CRC, thereby enhancing the antitumor immune response (32, 33). Additionally, B cells produce antibodies that can either directly neutralize tumor cells or mediate tumor cell killing through antibody-dependent cell-mediated cytotoxicity (ADCC) (34). However, it is important to recognize that B cells can sometimes have a detrimental effect on CRC. Research indicates that B cells within the CRC-TME can secrete growth factors that promote tumor growth and invasion (35). Moreover, B cells are involved in the formation of tertiary lymphoid structures (TLSs), which consist of CD20+ B cells, CD4+ follicular helper T cells, and follicular dendritic cells. A series of preclinical and clinical studies have found that TLS formation is associated with a lower risk of disease recurrence and improved prognosis, suggesting that TLSs play a significant role in CRC-related immune responses and disease progression (36, 37). Thus, the role of B cells in CRC growth and metastasis warrants careful consideration.

### 2.3 Macrophage

As a crucial subset of TME cells, tumor-associated macrophages (TAMs) primarily originate from monocytes in the bone marrow and play a significant role in the initiation, progression, and invasion of CRC (38, 39). The influence of TAMs on patients with CRC remains contentious, largely due to the distinctive characteristics of TAMs. As a highly heterogeneous and plastic cell population, TAMs interact with the TME in various ways depending on their subtype. Anfray et al. (40). identified at least two subtypes of TAMs within the TME: classically activated M1 macrophages and alternatively activated M2 macrophages. M1 macrophages exert antitumor effects primarily through the secretion of cytokines, including interleukin-6, interleukin-12, and tumor necrosis factor α (TNF-α), which directly kill tumor cells (41). In contrast, M2 macrophages play a role in tumor progression by contributing to basement membrane disruption and deposition, leukocyte accumulation, angiogenesis, and immune suppression (42). Additionally, macrophages are instrumental in remodeling the TME matrix, with the expression of various remodeling enzymes promoting an environment conducive to CRC growth. A study conducted with single-cell sequencing and spatial analysis of tumor and adjacent normal tissues from five non-metastatic patients found that interactions between fibroblasts and macrophages facilitate TME remodeling. This process contributes to the formation of a fibrotic microenvironment. Consequently, this remodeling prevents lymphocyte infiltration into the tumor core, thereby protecting CRC (43).

### 2.4 Neutrophils

Neutrophils are the most widely distributed cells in the innate immune system. They participate in tumor cell proliferation and suppress other immune cells. This involvement occurs through inflammatory signaling pathways, thereby exerting antitumor effects. In cancer, tumor-associated neutrophils (TANs) are classified into two phenotypes: N1 and N2 (44, 45). N1 neutrophils are considered to possess antitumor functions, while N2 neutrophils are associated with protumor activities. A meta-analysis evaluating the neutrophil-to-lymphocyte ratio (NLR) and TANs in relation to cancer prognosis has demonstrated that both NLR and TANs hold clinical promise as prognostic indicators of poor cancer outcomes (46). The role of neutrophils in CRC has garnered significant research interest, particularly regarding their formation of extracellular traps, known as neutrophil extracellular traps (NETs). Elevated levels of NETs are frequently observed both *in vivo* and *in vitro* in CRC patients and are closely associated with tumor recurrence and metastasis (47, 48). NETs often facilitate the adhesion of circulating tumor cells (CTCs) to tissue surfaces, thereby increasing the migration of CRC cells to critical areas of the body, such as the liver, lungs, and peritoneal cavity (49).

### 2.5 Natural killer cells

Natural killer (NK) cells, as the first line of defense in the innate immune system, play a crucial role in antitumor responses. A study found that a higher abundance of CD56-positive NK cells was significantly associated with prolonged overall survival in rectal cancer patients who underwent chemotherapy (50). However, NK cells are not solely enhancers of antitumor responses. NK cells can also promote the production of vascular endothelial growth factor (VEGF) and angiopoietins, indicating their role in facilitating angiogenesis (51, 52).

**Figure 1:**
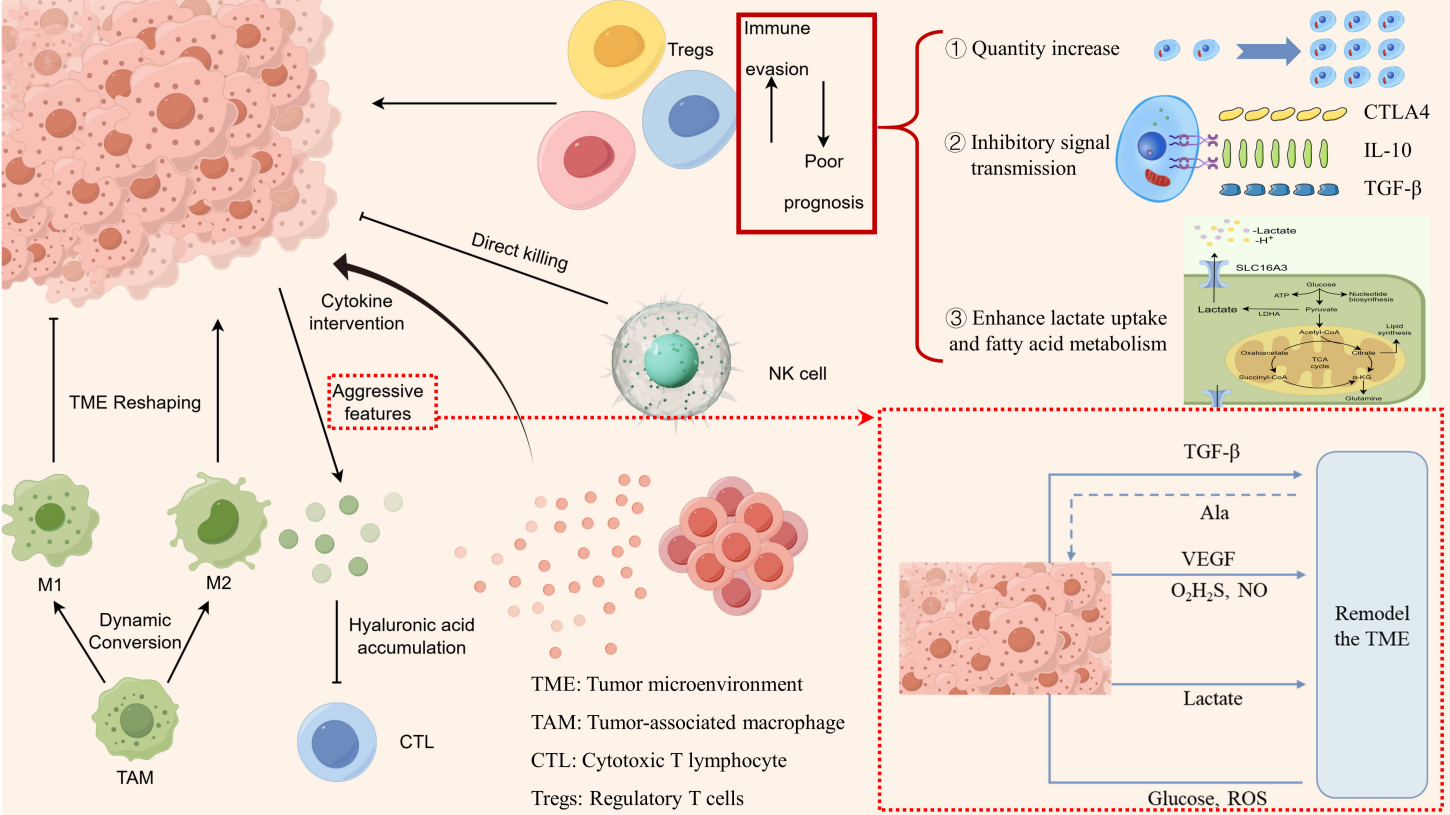
Characteristics of immune cell alterations in the immune microenvironment of colorectal cancer.

The immune system is a key component of the TME. Immune cells in CRC patients exhibit high plasticity, and changes in the cytokine milieu or metabolic conditions can influence the phenotype and function of these immune cells. Therefore, when evaluating the TME, it is crucial to consider the frequency and phenotypes of immune cells within the tumor. In summary, the significant impact of various immune cells on CRC progression is illustrated in **Figure 1**.

## 3 Characteristics of cellular pan-apoptosis in the immune microenvironment of colorectal cancer

**Figure 2:**
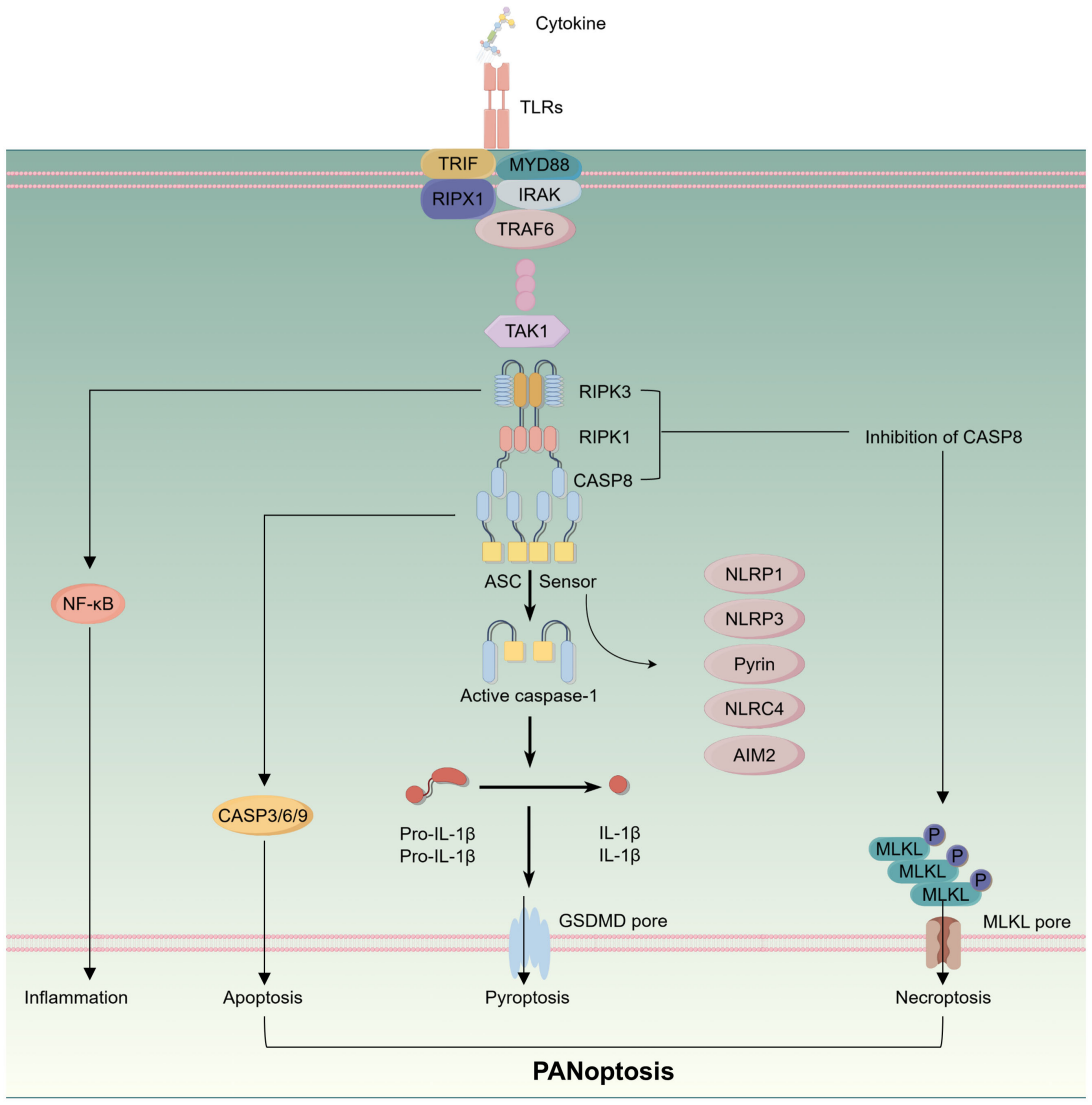
Schematic representation of intracellular signaling pathways in the process of panoptosis.

The concept of pan-apoptosis was first introduced by the Malireddi team, who identified a protein complex subsequently termed the PANoptosome, which can induce a distinct form of cell death (13). Pan-apoptosis encompasses features of pyroptosis, apoptosis, and necroptosis, involving various factors that are integral to immune responses. Its significance lies in driving innate immune responses and inflammation, with detailed signaling pathways illustrated in **Figure 2**. Consequently, pan-apoptosis plays a crucial role in tumor initiation and treatment (53). A study evaluating the expression of PANoptosome across 33 cancer types, alongside genomic, epigenomic, and prognostic analyses, found that elevated PANoptosis scores were closely associated with the infiltration levels of most immune cells within the TME and across various cancers. In other words, patients with high PANoptosis scores benefited from immunotherapy, exhibiting improved survival outcomes (54). It is currently widely accepted that pan-apoptosis is linked to the modulation of innate immune responses and inflammation and cannot be solely attributed to pyroptosis, apoptosis, or necroptosis in isolation. Therefore, it is essential to analyze the pan-apoptotic characteristics of various immune cells within the CRC immune microenvironment and their potential as therapeutic targets. The following discussion will elaborate on several immune cells that may be influenced by the occurrence of PANoptosis within the CRC immune microenvironment.

### 3.1 Characteristics of Pan-apoptosis in neutrophils

Neutrophils, the most prevalent type of leukocyte, are integral to the body’s initial defense against pathogenic invasion. Recent studies have elucidated their involvement in the mechanisms underlying PANoptosis, a novel form of cell death. The stimulator of interferon genes (STING) has emerged as a significant inducer of PANoptosis, facilitating immune responses directed toward tumors. One pivotal investigation revealed that the activation and dimerization of STING lead to the direct phosphorylation of TBK1 and IRF3, which subsequently results in the upregulation of apoptosis, pyroptosis, and necroptosis, ultimately culminating in pan-apoptosis (55). The TANs have drawn significant attention as inflammatory biomarkers within the TME. Evidence suggests that the pan-apoptosis of neutrophils is intricately linked to tumor progression and patient prognosis in various malignancies, such as lung cancer and thyroid cancer (56, 57). Specifically, in the context of non-small cell lung cancer, TANs that exhibit enhanced PANoptosis contribute to the immunosuppressive milieu within the TME, thereby promoting tumor growth. Understanding the multifaceted roles of TANs is essential for elucidating the mechanisms underlying CRC progression and prognosis. This knowledge opens promising avenues for the development of novel therapeutic strategies aimed at modulating neutrophil behavior in the TME.

### 3.2 Tumor-associated macrophages

PANoptosis has the capacity to directly induce tumor cell death, serving as a potential therapeutic target in cancer treatment. Moreover, a close relationship exists between innate immunity and PANoptosis, with the PANoptosome playing a crucial role in inflammatory immune responses, particularly evident in macrophages (58). Within the TME, TAMs primarily originate from bone marrow-derived monocytes, and the associated cell death pathways often exhibit intricate multi-level crosstalk. Similar to other cell types, the characteristics of PANoptosis in TAMs are influenced by three key genes: TAK1, CDK1, and SHARPIN. Transforming growth factor-beta-activated kinase 1 (TAK1) is a fundamental component of both innate and adaptive immune signaling and acts as a master regulator of PANoptosis. Research at the cellular level has revealed that macrophages with TAK1 deficiency exhibit necroptosis driven by the RIPK3-MLKL pathway, independent of RIPK1 kinase activity. *In vivo* studies indicate that inactivation of TAK1 leads to myeloid proliferation and a severe sepsis-like syndrome driven by the RIPK3-caspase-8 signaling axis (59).

In pancreatic ductal adenocarcinoma (PDAC), RNA sequencing and multiplex immunofluorescence have shown that early-stage liver metastatic patients (T1M1) exhibit increased expression of mixed lineage kinase domain-like pseudokinase (MLKL). These patients also demonstrate enhanced necroptotic pathways compared to non-metastatic patients (T1M0). This suggests that MLKL-driven necroptosis recruits macrophages, amplifying the tumor’s CD47 “don’t eat me” signal and inducing the formation of macrophage extracellular traps to activate CXCL8. CXCL8 further initiates epithelial-mesenchymal transition (EMT), ultimately supporting liver metastasis in PDAC (60). In CRC, therapeutic resistance is significantly influenced by the TME, where TAMs play a pivotal role. Oxaliplatin (OX), a third-generation platinum-based drug, is widely utilized as a first-line chemotherapy agent for CRC. However, M2-type TAMs serve as critical mediators of OX resistance. One study demonstrated that M2-TAMs confer OX resistance by enhancing METTL3-mediated m6A modification of cellular RNA. Targeting the necroptosis pathways in M2-TAMs presents a promising strategy to effectively mitigate OX resistance in CRC.

### 3.3 T cell

The cancer immune cycle provides a framework for understanding the mechanisms that activate T cell responses to tumors. However, this cycle is frequently constrained by T cell ignorance, a phenomenon induced by tumor-intrinsic immune editing, which obstructs the initiation and sustained engagement of adaptive immunity. Targeting PANoptosis could offer a novel approach to impede immune evasion, creating a positive feedback mechanism that enhances immune activation and helps counteract the immunoresistance observed in persistent tumors.

Recent investigations utilizing ultrasound nanomedicine combined with nano/gene-engineered extracellular vesicles have introduced innovative strategies for tumor immune re-editing. These approaches have demonstrated the ability to induce a highly immunogenic form of pan-apoptosis within tumors (61). Within the TME, PANoptosis stimulates antigen-presenting cells, promotes the cross-priming of CD8 T cells, and strengthens the overall anti-tumor immune response. Furthermore, T cell antigen receptors (TCRs) within the TME enable T cells to effectively identify both self and non-self antigens. This recognition process allows T cells to detect aberrant expression of endogenous proteins in cancer cells, influencing their differentiation and functional capabilities (62). Research focusing on melanoma has revealed that wild-type tumor cells can adapt to CTL attacks by modulating their mTOR signaling pathway. This adaptation involves shifting towards enhanced mTORC2 activity, which helps them evade apoptosis and necroptosis (63).

In CRC, tumor cells may leverage various signaling mechanisms to avoid inherent pan-apoptosis, thereby resisting attacks from T cells. This evasion likely plays a significant role in the dynamics of cancer immune responses and highlights the necessity of further exploring this pathway for developing novel therapeutic strategies within the field of immuno-oncology.

### 3.4 Others

PD-1 (Programmed Cell Death Protein 1) is an inhibitory receptor expressed on the surface of T cells and other immune cells, while PD-L1 (Programmed Cell Death Ligand 1) is predominantly expressed on tumor cells and certain immune cells. This pathway promotes immune evasion within the tumor microenvironment by suppressing T cell activity and proliferation. PANoptosis plays a significant role in regulating the PD-1/PD-L1 pathway, influencing immune checkpoint modulation (64). Pembrolizumab, a newer class of monoclonal antibody targeting PD-1, has been approved for the treatment of various cancers, including microsatellite instability-high (MSI-H) or mismatch repair-deficient (dMMR) CRC. This agent enhances anti-tumor immune responses by blocking the interaction between PD-1 and PD-L1, thereby ameliorating T cell apoptosis. Numerous chemotherapy agents, such as sorafenib, the frontline treatment for advanced hepatocellular carcinoma (HCC), can induce various forms of programmed cell death (PCD) including pyroptosis, apoptosis, and necroptosis. Sorafenib, in particular, has been shown to concurrently trigger all three PCD pathways, effectively leading to the elimination of tumor cells. Beyond its direct impact on tumor cells, PANoptosis exerts multifaceted, comprehensive, and sustained effects on immune cells. The signals generated by PANoptosis not only heighten the production and release of danger signals and chemokines but also act as an urgent call to action for the immune system. This call prompts the immune system to investigate, leading to enhanced immune cell migration, phagocytosis, antigen processing, MHC loading, maturation, and the cross-priming of T cells. This observation is consistent with findings that necroptotic cells can augment phagocyte-mediated cross-priming of CD8 T cells. Through these complex mechanisms, PANoptosis strengthens the capacity of immune cells to mount a robust and sustained immune response, not only enhancing direct cytotoxic effects on tumors but also amplifying systemic immune surveillance.

## 4 Conclusion

In CRC, the interplay of various forms of cell death enhances the process of PANoptosis, a complex mechanism that plays a role in tumor initiation, progression, and therapeutic responses under diverse conditions. This review underscores the critical importance of PANoptosis in regulating immune responses within the tTME. It is essential to recognize that PANoptosis may act as a double-edged sword in cancer treatment, potentially promoting the growth of cancer cells. The intricate nature of PANoptosis involves both genetic and epigenetic modifications. Additionally, one of the significant obstacles in utilizing PANoptosis therapeutically is the inconsistent expression and function of molecules associated with PANoptosomes, which can vary across different stages of CRC. Despite these challenges, progress in molecular, genetic, and epigenetic targeting and delivery systems offers promising possibilities for leveraging PANoptosis as an effective tool. Coupled with advancements in precision and personalized medicine, these developments can enhance CRC treatment outcomes.

This review also highlights the profound influence of the immune microenvironment in CRC on various immune cell types. Moreover, we examine the dual role of PANoptosis in developing future therapeutic approaches for CRC. Identifying key regulators of PANoptosis and comprehending the underlying mechanisms are crucial, as these insights could pave the way for new targeted and personalized treatment options for patients.

## References

[1] Bray F Laversanne M Sung H Ferlay J Siegel RL Soerjomataram I. Global cancer statistics 2022: GLOBOCAN estimates of incidence and mortality worldwide for 36 cancers in 185 countries. CA: Cancer J Clin. (2024) 74:229–63. doi: 10.3322/caac.21834 38572751

[2] Ma Y Li J Zhao X Ji C Hu W Ma Y. Multi-omics cluster defines the subtypes of CRC with distinct prognosis and tumor microenvironment. Eur J Med Res. (2024) 29:207. doi: 10.1186/s40001-024-01805-8 38549156 PMC10976740

[3] Ajebli M Meretsky CR Akdad M Amssayef A Hebi M. The role of dietary vitamins and antioxidants in preventing colorectal cancer: A systematic review. Cureus. (2024) 16:e64277. doi: 10.7759/cureus.64277 39130946 PMC11315617

[4] Xu Z Zheng J Chen Z Guo J Li X Wang X. Multilevel regulation of Wnt signaling by Zic2 in colon cancer due to mutation of β-catenin. Cell Death disease. (2021) 12:584. doi: 10.1038/s41419-021-03863-w 34099631 PMC8184991

[5] Liu Z Wang L Guo C Liu L Jiao D Sun Z. TTN/OBSCN ‘Double-Hit’ predicts favourable prognosis, ‘immune-hot’ subtype and potentially better immunotherapeutic efficacy in colorectal cancer. J Cell Mol Med. (2021) 25:3239–51. doi: 10.1111/jcmm.16393 PMC8034451 33624434

[6] Hailun X Huang S Yuan G Tang S Gan J. Prognostic significance of preoperative fibrinogen-to-prealbumin ratio in patients with stage I-III colorectal cancer undergoing surgical resection: A retrospective cohort study. BioMed Res Int. (2021) 2021:3905353. doi: 10.1155/2021/3905353 33521127 PMC7817313

[7] Isik Z Leblebici A Demir Karaman E Karaca C Ellidokuz H Koc A. In silico identification of novel biomarkers for key players in transition from normal colon tissue to adenomatous polyps. PloS One. (2022) 17:e0267973. doi: 10.1371/journal.pone.0267973 35486660 PMC9053805

[8] Bortolomeazzi M Keddar MR Montorsi L Acha-Sagredo A Benedetti L Temelkovski D. Immunogenomics of colorectal cancer response to checkpoint blockade: analysis of the KEYNOTE 177 trial and validation cohorts. Gastroenterology. (2021) 161:1179–93. doi: 10.1053/j.gastro.2021.06.064 PMC8527923 34197832

[9] Chen X Poetsch A. The role of cdo1 in ferroptosis and apoptosis in cancer. Biomedicines. (2024) 12:918. doi: 10.3390/biomedicines12040918 38672271 PMC11047957

[10] He N Li D Xu F Jin J Li L Tian L. LncPCD: a manually curated database of experimentally supported associations between lncRNA-mediated programmed cell death and diseases. Database: J Biol Database Curation. (2023) baad087. doi: 10.1093/database/baad087 PMC10681436 38011720

[11] Peng F Liao M Qin R Zhu S Peng C Fu L. Regulated cell death (RCD) in cancer: key pathways and targeted therapies. Signal transduct target Ther. (2022) 7:286. doi: 10.1038/s41392-022-01110-y 35963853 PMC9376115

[12] Garg AD Agostinis P. Cell death and immunity in cancer: From danger signals to mimicry of pathogen defense responses. Immunol Rev. (2017) 280:126–48. doi: 10.1111/imr.12574 29027218

[13] Malireddi RKS Kesavardhana S Kanneganti TD. ZBP1 and TAK1: master regulators of NLRP3 inflammasome/pyroptosis, apoptosis, and necroptosis (PAN-optosis). Front Cell infect Microbiol. (2019) 9:406. doi: 10.3389/fcimb.2019.00406 31850239 PMC6902032

[14] Yin X Zhang H Wang J Bian Y Jia Q Yang Z. lncRNA FLJ20021 regulates CDK1-mediated PANoptosis in a ZBP1-dependent manner to increase the sensitivity of laryngeal cancer-resistant cells to cisplatin. Discover Oncol. (2024) 15:265. doi: 10.1007/s12672-024-01134-6 PMC11226695 38967843

[15] Zhang CC Li CG Wang YF Xu LH He XH Zeng QZ. Chemotherapeutic paclitaxel and cisplatin differentially induce pyroptosis in A549 lung cancer cells via caspase-3/GSDME activation. Apoptosis: an Int J programmed Cell death. (2019) 24:312–25. doi: 10.1007/s10495-019-01515-1 30710195

[16] Chen S Peng A Chen M Zhan M. Nanomedicines targeting activation of STING to reshape tumor immune microenvironment and enhance immunotherapeutic efficacy. Front Oncol. (2022) 12:1093240. doi: 10.3389/fonc.2022.1093240 36741735 PMC9890065

[17] Qu L Ding S Long Q Zheng S Chen ZS Yi W. Editorial: DNA methylation, tumor microenvironment and their effects in immunotherapy and drug resistance in thoracic tumors. Front Immunol. (2024) 15:1357278. doi: 10.3389/fimmu.2024.1357278 38288309 PMC10822968

[18] Li B Liu X Wu G Liu J Cai S Wang F. MicroRNA-934 facilitates cell proliferation, migration, invasion and angiogenesis in colorectal cancer by targeting B-cell translocation gene 2. Bioengineered. (2021) 12:9507–19. doi: 10.1080/21655979.2021.1996505 PMC8809948 34699325

[19] Liu GH Chen T Zhang X Ma XL Shi HS. Small molecule inhibitors targeting the cancers. MedComm. (2022) 3:e181. doi: 10.1002/mco2.181 36254250 PMC9560750

[20] Gao SH Liu SZ Wang GZ Zhou GB. CXCL13 in cancer and other diseases: biological functions, clinical significance, and therapeutic opportunities. Life (Basel Switzerland). (2021) 11:1282. doi: 10.3390/life11121282 34947813 PMC8708574

[21] Khaliq AM Erdogan C Kurt Z Turgut SS Grunvald MW Rand T. Refining colorectal cancer classification and clinical stratification through a single-cell atlas. Genome Biol. (2022) 23:113. doi: 10.1186/s13059-022-02677-z 35538548 PMC9092724

[22] Su ZY Siak PY Leong CO Cheah SC. Nasopharyngeal carcinoma and its microenvironment: past, current, and future perspectives. Front Oncol. (2022) 12:840467. doi: 10.3389/fonc.2022.840467 35311066 PMC8924466

[23] Shao Y Li R Chen G Zhang L. Pan-cancer analysis of GALNT6 with potential implications for prognosis and tumor microenvironment in human cancer based on bioinformatics and qPCR verification. Int J Gen Med. (2024) 17:2187–201. doi: 10.2147/ijgm.S459953 PMC11104441 38770365

[24] Luo P Zhou P. The relationship between PBLS and osteosarcoma distribution in different subgroups and the survival and prognosis of osteosarcoma. J Oncol. (2023) 2023:3893134. doi: 10.1155/2023/3893134 37064862 PMC10104740

[25] Tang L He S Yin Y Liu H Hu J Cheng J. Combination of nanomaterials in cell-based drug delivery systems for cancer treatment. Pharmaceutics. (2021) 13:1888. doi: 10.3390/pharmaceutics13111888 34834304 PMC8621332

[26] Martinez-Ordoñez A Duran A Ruiz-Martinez M Cid-Diaz T Zhang X Han Q. Hyaluronan driven by epithelial aPKC deficiency remodels the microenvironment and creates a vulnerability in mesenchymal colorectal cancer. Cancer Cell. (2023) 41:252–271.e9. doi: 10.1016/j.ccell.2022.11.016 36525970 PMC9931663

[27] Zafari N Khosravi F Rezaee Z Esfandyari S Bahiraei M Bahramy A. The role of the tumor microenvironment in colorectal cancer and the potential therapeutic approaches. J Clin Lab anal. (2022) 36:e24585. doi: 10.1002/jcla.24585 35808903 PMC9396196

[28] Wan J Wu Y Huang L Tian Y Ji X Abdelaziz MH. ILC2-derived IL-9 inhibits colorectal cancer progression by activating CD8(+) T cells. Cancer letters. (2021) 502:34–43. doi: 10.1016/j.canlet.2021.01.002 33429004

[29] Tosolini M Kirilovsky A Mlecnik B Fredriksen T Mauger S Bindea G. Clinical impact of different classes of infiltrating T cytotoxic and helper cells (Th1, th2, treg, th17) in patients with colorectal cancer. Cancer Res. (2011) 71:1263–71. doi: 10.1158/0008-5472.Can-10-2907 21303976

[30] Chen Q Shen M Yan M Han X Mu S Li Y. Targeting tumor-infiltrating CCR8(+) regulatory T cells induces antitumor immunity through functional restoration of CD4(+) T(convs) and CD8(+) T cells in colorectal cancer. J Trans Med. (2024) 22:709. doi: 10.1186/s12967-024-05518-8 PMC11290082 39080766

[31] Kuwahara T Hazama S Suzuki N Yoshida S Tomochika S Nakagami Y. Intratumoural-infiltrating CD4 + and FOXP3 + T cells as strong positive predictive markers for the prognosis of resectable colorectal cancer. Br J cancer. (2019) 121:659–65. doi: 10.1038/s41416-019-0559-6 PMC6889292 31488881

[32] de Vries NL Swets M Vahrmeijer AL Hokland M Kuppen PJ. The immunogenicity of colorectal cancer in relation to tumor development and treatment. Int J Mol Sci. (2016) 17:1030. doi: 10.3390/ijms17071030 27367680 PMC4964406

[33] Atreya I Neurath MF. How the tumor micromilieu modulates the recruitment and activation of colorectal cancer-infiltrating lymphocytes. Biomedicines. (2022) 10:2940. doi: 10.3390/biomedicines10112940 36428508 PMC9687992

[34] Saudi A Banday V Zirakzadeh AA Selinger M Forsberg J Holmbom M. Immune-activated B cells are dominant in prostate cancer. Cancers. (2023) 15:920. doi: 10.3390/cancers15030920 36765877 PMC9913271

[35] Wang SS Liu W Ly D Xu H Qu L Zhang L. Tumor-infiltrating B cells: their role and application in anti-tumor immunity in lung cancer. Cell Mol Immunol. (2019) 16:6–18. doi: 10.1038/s41423-018-0027-x 29628498 PMC6318290

[36] Alturaiki W Alkadi H Alamri S Awadalla ME Alfaez A Mubarak A. Association between the expression of toll-like receptors, cytokines, and homeostatic chemokines in SARS-CoV-2 infection and COVID-19 severity. Heliyon. (2023) 9:e12653. doi: 10.1016/j.heliyon.2022.e12653 36589720 PMC9788851

[37] Maurya NS Kushwah S Kushwaha S Chawade A Mani A. Prognostic model development for classification of colorectal adenocarcinoma by using machine learning model based on feature selection technique boruta. Sci Rep. (2023) 13:6413. doi: 10.1038/s41598-023-33327-4 37076536 PMC10115869

[38] Wang H Tian T Zhang J. Tumor-associated macrophages (TAMs) in colorectal cancer (CRC): from mechanism to therapy and prognosis. Int J Mol Sci. (2021) 22:8470. doi: 10.3390/ijms22168470 34445193 PMC8395168

[39] Wei C Yang C Wang S Shi D Zhang C Lin X. Crosstalk between cancer cells and tumor associated macrophages is required for mesenchymal circulating tumor cell-mediated colorectal cancer metastasis. Mol cancer. (2019) 18:64. doi: 10.1186/s12943-019-0976-4 30927925 PMC6441214

[40] Anfray C Ummarino A Andón FT Allavena P. Current strategies to target tumor-associated-macrophages to improve anti-tumor immune responses. Cells. (2019) 9:46. doi: 10.3390/cells9010046 31878087 PMC7017001

[41] Popēna I Ābols A Saulīte L Pleiko K Zandberga E Jēkabsons K. Effect of colorectal cancer-derived extracellular vesicles on the immunophenotype and cytokine secretion profile of monocytes and macrophages. Cell commun signal: CCS. (2018) 16:17. doi: 10.1186/s12964-018-0229-y 29690889 PMC5937830

[42] Zhu X Liang R Lan T Ding D Huang S Shao J. Tumor-associated macrophage-specific CD155 contributes to M2-phenotype transition, immunosuppression, and tumor progression in colorectal cancer. J immunother Cancer. (2022) 10:e004219. doi: 10.1136/jitc-2021-004219 36104099 PMC9476138

[43] Qi J Sun H Zhang Y Wang Z Xun Z Li Z. Single-cell and spatial analysis reveal interaction of FAP(+) fibroblasts and SPP1(+) macrophages in colorectal cancer. Nat Commun. (2022) 13:1742. doi: 10.1038/s41467-022-29366-6 35365629 PMC8976074

[44] Li K Shi H Zhang B Ou X Ma Q Chen Y. Myeloid-derived suppressor cells as immunosuppressive regulators and therapeutic targets in cancer. Signal transduct target Ther. (2021) 6:362. doi: 10.1038/s41392-021-00670-9 34620838 PMC8497485

[45] Qiao Q Hu S Wang X. The regulatory roles and clinical significance of glycolysis in tumor. Cancer Commun (London England). (2024) 44:761–86. doi: 10.1002/cac2.12549 PMC11260772 38851859

[46] Cupp MA Cariolou M Tzoulaki I Aune D Evangelou E Berlanga-Taylor AJ. Neutrophil to lymphocyte ratio and cancer prognosis: an umbrella review of systematic reviews and meta-analyses of observational studies. BMC Med. (2020) 18:360. doi: 10.1186/s12916-020-01817-1 33213430 PMC7678319

[47] Richardson JJR Hendrickse C Gao-Smith F Thickett DR. Neutrophil extracellular trap production in patients with colorectal cancer in vitro. Int J Inflammation. (2017) 2017:4915062. doi: 10.1155/2017/4915062 PMC5554570 28828191

[48] Shang A Gu C Zhou C Yang Y Chen C Zeng B. Exosomal KRAS mutation promotes the formation of tumor-associated neutrophil extracellular traps and causes deterioration of colorectal cancer by inducing IL-8 expression. Cell commun signal: CCS. (2020) 18:52. doi: 10.1186/s12964-020-0517-1 32228650 PMC7106821

[49] Najumudeen AK Ceteci F Fey SK Hamm G Steven RT Hall H. The amino acid transporter SLC7A5 is required for efficient growth of KRAS-mutant colorectal cancer. Nat Genet. (2021) 53:16–26. doi: 10.1038/s41588-020-00753-3 33414552

[50] Alderdice M Dunne PD Cole AJ O’Reilly PG McArt DG Bingham V. Natural killer-like signature observed post therapy in locally advanced rectal cancer is a determinant of pathological response and improved survival. Modern Pathol. (2017) 30:1287–98. doi: 10.1038/modpathol.2017.47 28621318

[51] Bald T Krummel MF Smyth MJ Barry KC. The NK cell-cancer cycle: advances and new challenges in NK cell-based immunotherapies. Nat Immunol. (2020) 21:835–47. doi: 10.1038/s41590-020-0728-z PMC8406687 32690952

[52] Wang J Liu X Jin T Cao Y Tian Y Xu F. NK cell immunometabolism as target for liver cancer therapy. Int immunopharmacol. (2022) 112:109193. doi: 10.1016/j.intimp.2022.109193 36087507

[53] Karki R Sundaram B Sharma BR Lee S Malireddi RKS Nguyen LN. ADAR1 restricts ZBP1-mediated immune response and PANoptosis to promote tumorigenesis. Cell Rep. (2021) 37:109858. doi: 10.1016/j.celrep.2021.109858 34686350 PMC8853634

[54] Zhuang L Sun Q Huang S Hu L Chen Q. A comprehensive analysis of PANoptosome to prognosis and immunotherapy response in pan-cancer. Sci Rep. (2023) 13:3877. doi: 10.1038/s41598-023-30934-z 36890219 PMC9995449

[55] Messaoud-Nacer Y Culerier E Rose S Maillet I Rouxel N Briault S. STING agonist diABZI induces PANoptosis and DNA mediated acute respiratory distress syndrome (ARDS). Cell Death disease. (2022) 13:269. doi: 10.1038/s41419-022-04664-5 35338116 PMC8953969

[56] Li Y Wu D. Identification of signature genes and immune infiltration analysis in thyroid cancer based on PANoptosis related genes. Front endocrinol. (2024) 15:1397794. doi: 10.3389/fendo.2024.1397794 PMC11298382 39104814

[57] Hu Q Wang R Zhang J Xue Q Ding B. Tumor-associated neutrophils upregulate PANoptosis to foster an immunosuppressive microenvironment of non-small cell lung cancer. Cancer immunol immunother: CII. (2023) 72:4293–308. doi: 10.1007/s00262-023-03564-7 PMC10992448 37907644

[58] Zhu P Ke ZR Chen JX Li SJ Ma TL Fan XL. Advances in mechanism and regulation of PANoptosis: Prospects in disease treatment. Front Immunol. (2023) 14:1120034. doi: 10.3389/fimmu.2023.1120034 36845112 PMC9948402

[59] Malireddi RKS Gurung P Kesavardhana S Samir P Burton A Mummareddy H. Innate immune priming in the absence of TAK1 drives RIPK1 kinase activity-independent pyroptosis, apoptosis, necroptosis, and inflammatory disease. J Exp Med. (2020) 217:jem.20191644. doi: 10.1084/jem.20191644 31869420 PMC7062518

[60] Liao CY Li G Kang FP Lin CF Xie CK Wu YD. Necroptosis enhances ‘don’t eat me’ signal and induces macrophage extracellular traps to promote pancreatic cancer liver metastasis. Nat Commun. (2024) 15:6043. doi: 10.1038/s41467-024-50450-6 39025845 PMC11258255

[61] Zhou L Lyu J Liu F Su Y Feng L Zhang X. Immunogenic PANoptosis-initiated cancer sono-immune reediting nanotherapy by iteratively boosting cancer immunity cycle. Adv materials (Deerfield Beach Fla). (2024) 36:e2305361. doi: 10.1002/adma.202305361 37699593

[62] Liu ZY Zheng M Li YM Fan XY Wang JC Li ZC. RIP3 promotes colitis-associated colorectal cancer by controlling tumor cell proliferation and CXCL1-induced immune suppression. Theranostics. (2019) 9:3659–73. doi: 10.7150/thno.32126 PMC6587173 31281505

[63] Lin CP Traets JJH Vredevoogd DW Visser NL Peeper DS. TSC2 regulates tumor susceptibility to TRAIL-mediated T-cell killing by orchestrating mTOR signaling. EMBO J. (2023) 42:e111614. doi: 10.15252/embj.2022111614 36715448 PMC9975943

[64] Maitra S Bhattacharya D Paul S Chowdhury PG Mandal D Haldar PK. Programmed cell death protein 1 (PD-1) in relation to PANoptosis: immune pharmacological targets for management of breast adenocarcinoma. Endocr Metab Immune Disord Drug targets. (2023) 23:1571–85. doi: 10.2174/1871530323666230213121803 36788687

